# Stealth Materials Based on Laser-Induced Graphene: Developments and Challenges

**DOI:** 10.3390/nano15080623

**Published:** 2025-04-18

**Authors:** Xinjian Lu, Ruige Su, Guiyong Chen, Wenxin Li, Misheng Liang, Rui You

**Affiliations:** 1 AVIC Chengdu Aircraft Industrial (Group) Co., Ltd., Chengdu 610092, China; 15976854401@163.com (X.L.); cgybuaa@126.com (G.C.); m15850565816@163.com (W.L.); 2 Laboratory of the Intelligent Microsystem, Beijing Information Science and Technology University, Beijing 100192, China; ruigesu@163.com

**Keywords:** laser-induced graphene, electromagnetic interference, multispectral absorbing material, broadband absorbing material, metasurface

## Abstract

Laser-induced graphene (LIG) has become a promising stealth material due to its excellent electromagnetic loss characteristics in the terahertz and microwave bands (2–18 Ghz) and the advantages of low-cost large-scale manufacturing. With the rapid advancement of electromagnetic detection technologies toward multispectral and high-dynamic-range capabilities, there is an increasing demand for LIG-based stealth materials with superior absorption performance. The synergistic design of functional material doping and structural configurations has been identified as a critical approach to achieve high electromagnetic shielding performance in LIG-based composites. This article briefly reviews the developmental progress of LIG-based electromagnetic stealth materials, with a particular emphasis on doping technologies and shielding mechanisms tailored for stealth applications. Furthermore, we propose potential future development pathways for LIG-based stealth materials to facilitate their transition toward broader practical applications.

## 1. Introduction

With the high-frequency and integrated development of modern electronic devices, as well as the iterative upgrades in military stealth technologies, research on electromagnetic shielding materials has transcended traditional functional boundaries and is evolving toward intelligence and being lightweight [1,2,3,4]. In cutting-edge application fields such as electromagnetic interference (EMI) resistance, radar wave absorption, and multifunctional stealth, materials are required not only to possess excellent electromagnetic wave attenuation capabilities but also to meet the demands of impedance matching under complex working conditions, structural adaptability, and multifunctional integration [5,6,7,8,9]. This development trend has posed fundamental challenges to traditional metal-based shielding materials: their shielding principle relying on high-reflection mechanisms leads to secondary electromagnetic pollution; the weight issues caused by dense structures limit aerospace applications; and complex processing techniques struggle to meet the integration requirements of flexible electronic devices [10,11,12,13,14]. In this context, laser-induced graphene (LIG), as a revolutionary carbon-based material, is driving a paradigm shift in next-generation electromagnetic shielding materials [15,16,17,18].

The breakthrough advantages of LIG stem from its unique three-dimensionally interconnected porous network structure [19,20,21]. By precisely controlling laser parameters, a conductive network with hierarchical porosity and ultrahigh specific surface area can be directly fabricated on polymer substrates such as polyimide [22,23,24]. This structure exhibits triple synergistic effects in dielectric properties: First, the gradient-distributed porous structure significantly improves the electrical impedance matching at the air/material interface, enabling incident electromagnetic waves to efficiently couple into the material’s interior rather than being reflected at the surface. Second, the three-dimensional conductive network converts electromagnetic energy into thermal energy through interfacial polarization, dipole relaxation, and multilevel scattering mechanisms. Third, the abundant grain boundaries and defect states within the material act as resonant cavities for electromagnetic waves, triggering multiple reflection interference effects [25]. Additionally, the LIG manufacturing process offers significant advantages, including simplified processing procedures, environmental friendliness, and compliance with industrial requirements for low-cost, high-efficiency production [26,27,28,29,30]. These merits make LIG the most promising candidate material for next-generation electromagnetic shielding.

In recent years, as performance requirements for electromagnetic functional materials continue to escalate in advanced fields such as military stealth coatings, aerospace thermal–electromagnetic synergistic protection systems, and quantum communication devices, traditional electromagnetic regulation technologies are facing unprecedented multidimensional challenges [31,32,33,34,35]. In scenarios including fifth-generation fighter radar stealth, sixth-generation communication base station anti-interference, and deep-space detector radiation protection, materials must not only achieve core functionalities such as cross-band compatibility and ultra-broadband absorption but also exhibit dynamic tunability to adapt to complex electromagnetic environments. This evolving demand has exposed significant limitations in single-component LIG materials, prompting researchers to shift toward constructing LIG-based multifunctional composite systems. However, the introduction of functional additives complicates the electromagnetic wave absorption mechanisms of LIG-based composites, as key parameters—including conductivity, permeability, porosity, and defect density—become closely interdependent. Notably, decoupling these interdependent factors remains a critical challenge. This perspective article systematically reviews the electromagnetic shielding mechanisms of LIG-based composites and synthesizes key influencing factors identified in current research, aiming to provide strategic guidance for designing LIG composites tailored to diverse application requirements.

## 2. Electromagnetic Shielding Mechanism of Three-Dimensional Porous Conducting Structures Represented by Laser-Induced Graphene

According to Schelkunoff’s theory, when electromagnetic waves propagate to the surface of a shielding material, three distinct attenuation mechanisms typically occur: (i) reflection loss (SE_R_) at the incident interface between air and the shielding layer; (ii) absorption loss (SE_A_) caused by electromagnetic waves penetrating the shielding layer without reflection; and (iii) multiple reflection loss (SE_M_) arising from internal interfacial reflections within the shielding layer. The total electromagnetic interference shielding effectiveness (EMI SE), denoted as SE_T_, as shown in Equation (1), is the sum of these three parts.

(1) $${SE}_{T}\left(dB\right)={SE}_{R}+{SE}_{A}+{SE}_{M}$$

Both reflection loss and absorption loss are inherently related to the electrical conductivity (σ) and magnetic permeability (μ) of the shielding material. Absorption loss (SE_A_) and reflection loss (SE_R_) can be expressed by Equations (2) and (3), respectively [36,37].

(2) $${SE}_{A}\left(dB\right)=8.7\frac{t}{\delta }=8.7t\sqrt{\pi f\mu \sigma }$$

(3) $${SE}_{R}\left(dB\right)=39.5+10\log\frac{\sigma }{2\pi f\mu }$$

where f denotes the frequency of the incident electromagnetic wave. Generally, a higher electrical conductivity enhances reflection attenuation. To achieve a satisfactory shielding performance, the conductivity must reach at least 1 S/m [11]. From this perspective, metals such as gold, silver, and copper are excellent electromagnetic wave reflectors. However, high conductivity is not the sole criterion for EMI reflection loss, as μ and f also play critical roles. The excessive pursuit of high conductivity may exacerbate secondary reflection pollution. Consequently, strategies that endow conductive EMI shielding materials with tailored magnetic properties can effectively reduce reflection loss, thereby mitigating secondary electromagnetic pollution.

**Figure 1:**
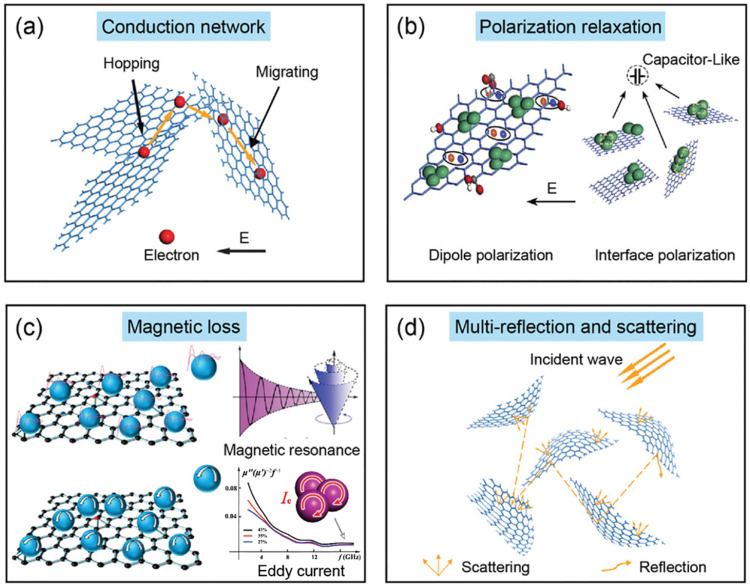
Electromagnetic loss mechanism of graphene and its derived materials [11].

Absorption loss primarily arises from the interaction of electromagnetic waves with electric dipoles or magnetic dipoles within the shielding layer, governed by the shielding material’s high electrical conductivity (Ohmic loss), substantial dielectric constant (dielectric loss), and elevated magnetic permeability (magnetic loss). The electromagnetic wave energy is ultimately converted into thermal energy through absorption loss and dissipation within the shielding layer. As shown in Figure 1, for graphene and its derivative materials, their enhanced electromagnetic properties predominantly govern electromagnetic wave absorption loss, with mechanisms primarily including conductive loss (from free charge carriers), polarization loss (dipole and interfacial polarization), magnetic loss (natural resonance and eddy current effects), and multiple scattering/reflection within the material’s hierarchical structure [11].

Based on the fundamental mechanisms and equations discussed above, the following critical aspects should be prioritized when designing LIG-based composite functional materials for electromagnetic wave absorption systems.

### 2.1. Electrical Conductivity and Ohmic Loss

As indicated by the formulas for absorption and reflection losses, increasing electrical conductivity enhances both reflection loss (SE_R_) and absorption loss (SE_A_), thereby dominantly determining the overall EMI shielding performance. Consequently, numerous studies have incorporated metallic nanoparticles (NPs) into the LIG conductive network through deposition or spraying techniques to elevate conductivity for superior shielding capabilities. However, it is crucial to note that the excessive pursuit of high conductivity not only exacerbates electromagnetic reflection pollution (as previously discussed) but also incurs higher costs and compromises the material’s mechanical–electromagnetic integrated performance. Thus, optimizing conductivity necessitates a balanced approach considering practical application requirements and cost-effectiveness.

### 2.2. Magnetic Permeability and Magnetic Loss

According to Equations (2) and (3), increasing magnetic permeability enhances SE_A_ while reducing SE_R_. Magnetic loss primarily originates from eddy current effects, natural resonance, and exchange resonance, which collectively convert electromagnetic energy into thermal dissipation. Introducing magnetic properties into conductive EMI shielding materials can effectively reduce reflection loss, thereby alleviating secondary electromagnetic pollution. Furthermore, the magnetoelectric coupling effects arising from the integration of dielectric and magnetic components in composites enable simultaneous optimization of impedance matching and attenuation capacity. Recent studies have demonstrated that loading magnetic metal oxide nanoparticles onto the 3D LIG conductive network allows the precise modulation of complex permittivity (ε, r) and permeability (μ, r), achieving desirable impedance matching [36,37,38,39]. This synergistic regulation of electrical and magnetic properties triggers multi-relaxation processes, magnetic resonance, electron hopping, eddy currents, and multi-scattering effects. These cooperative mechanisms facilitate exceptional electromagnetic wave absorption performance with broad bandwidth, positioning LIG-based composites as advanced solutions for next-generation electromagnetic shielding applications.

### 2.3. Porosity and Multiple Reflections/Scattering

As established, unreflected electromagnetic waves penetrate the shielding material and are dissipated by interactions with electric and magnetic dipoles, contributing to absorption loss. In three-dimensional porous conductive networks like LIG, electromagnetic waves undergo multiple reflections/scattering at the abundant interfaces of internal pore walls. These repeated interactions significantly prolong the energy propagation path, enabling the efficient conversion of wave energy into heat through coupled electrical–magnetic dissipation mechanisms. Notably, the porous architecture primarily enhances absorption dissipation rather than reflection, as the trapped waves are progressively attenuated within the hierarchical pore structure.

### 2.4. Defects/Doping and Polarization Loss

Defect engineering, encompassing lattice defects and heteroatom doping, has emerged as a strategic approach to tailor dielectric properties, achieving optimal synergy between impedance matching and loss capabilities. Inherent to the laser-induced synthesis mechanism, LIG inherently contains numerous disordered defects. While these defects moderately impair charge carrier transport efficiency (affecting conductivity), they simultaneously amplify polarization loss by creating localized charge imbalance regions. Experimental studies confirm that laser processing parameters critically influence defect density in LIG, with these defect sites acting as polarization centers under alternating electromagnetic fields, thereby enhancing EMI shielding performance [40,41]. Beyond intrinsic defects, heteroatom doping (e.g., nitrogen, sulfur) or the formation of heterogeneous interfaces (e.g., with MXenes or ferrites) can substantially strengthen interfacial polarization, further promoting electromagnetic wave attenuation. This defect-mediated polarization mechanism, coupled with optimized conductivity and permeability, provides a multi-scale strategy for designing high-performance LIG-based absorbers with tunable electromagnetic dissipation properties.

### 2.5. Multilayer Structure Dissipation

At the macroscopic scale, LIG-based materials have been assembled into components with meticulously engineered geometries to achieve efficient electromagnetic wave absorption and low-reflectivity shielding. Notably, electromagnetic metamaterials with gradient impedance structures and multilayered stacked architectures have garnered significant attention. These designs feature progressively increasing conductivity along the electromagnetic wave propagation direction, thereby enhancing both impedance matching and wave attenuation performance. Asymmetric geometries with tailored transitional shapes can induce impedance gradients that promote the deep penetration of incident waves into the material while minimizing surface reflection. Consequently, such periodic architectures have been engineered as energy-dissipative metamaterials to efficiently attenuate wave energy. Among various geometric configurations, pyramidal or wedge-shaped absorbers demonstrate superior broadband absorption characteristics, as their structural profiles enable gradual wave dissipation along the macroscopic geometry while suppressing reflectivity. Collectively, multilayered directional porous structures leverage two synergistic mechanisms: “Absorption–reflection–reabsorption” dynamics driven by gradient conductivity designs, which progressively trap and attenuate electromagnetic waves; Multi-component interfacial polarization and magnetoelectric coupling effects, which enhance energy dissipation through heterogeneous interfaces and hybrid loss mechanisms. These hierarchical architectures effectively block electromagnetic wave penetration while ensuring internal energy dissipation, positioning them as advanced solutions for high-performance, reflection-suppressed electromagnetic shielding applications.

## 3. Recent Research on LIG-Based Electromagnetic Stealth Materials

### 3.1. LIG-Based Multispectral Absorbing Material

**Figure 2:**
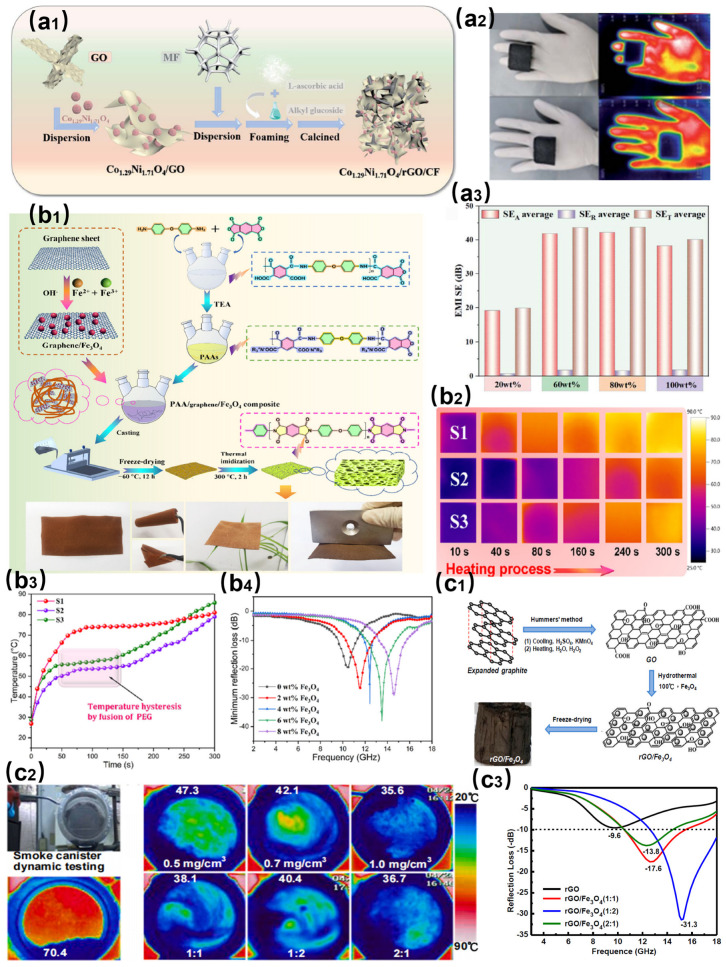
(**a1**) Synthesis method of Co_1.29_Ni_1.71_O_4_/rGO/CF composite foam. (**a2**) Infrared stealth capability of composite foam. (**a3**) Electromagnetic interference shielding performance of composite foam [42]. (**b1**) Scheme for the preparation of polyimide/graphene/Fe_3_O_4_ nanocomposites. (**b2**) Infrared camouflage of polyimide/graphene/Fe_3_O_4_ nanocomposites. (**b3**) Electromagnetic shielding properties of polyimide/graphene/Fe_3_O_4_ nanocomposites. (**b4**) Electromagnetic absorption properties of polyimide/graphene/Fe_3_O_4_ nanocomposites [43]. (**c1**) Synthesis strategy of porous rGO/Fe_3_O_4_ structures. (**c2**) Infrared stealth properties of porous rGO/Fe_3_O_4_. (**c3**) Evaluation of electromagnetic wave attenuation properties of porous rGO/Fe_3_O_4_ [44].

Recent breakthroughs in multifunctional stealth materials have primarily relied on microstructural design and multi-component synergistic strategies to achieve integrated optimization of electromagnetic wave absorption and infrared radiation regulation. As shown in Figure 2(a1), Shi et al. developed a dual three-dimensional continuous phase (d-3D-CP) structured Co_1.29_Ni_1.71_O_4_/rGO/CF composite foam via a foaming–calcination process [42]. As shown in Figure 2(a2), the synergistic effects of its 3D network and superhydrophobic properties (contact angle > 150°) enabled a stable infrared stealth performance at 56 °C and all-weather protection. As can be seen from Figure 2(a3), the EMI values of Co_1.29_Ni_1.71_O_4_/rGO/CF−60 wt%, −80 wt%, and −100 wt% exceed 30 dB throughout the X- and Ku-bands, which exhibit efficient EMI shielding performance.

As shown in Figure 2(b1), Wang et al. innovatively integrated phase-change material PEG (latent heat: 158 J/g) with electromagnetic loss components in polyimide/graphene/Fe_3_O_4_ hybrid aerogel-based composite films [43]. As shown in Figure 2(b2–b4), fabricated through freeze-drying and vacuum impregnation techniques, the flexible films exhibited a reflection loss of −38.5 dB in the 7.0–16.5 GHz range while maintaining temperature fluctuations below 5 °C via phase-change thermal buffering, validating the effectiveness of structure–function integration in electromagnetic–infrared bi-stealth applications. As shown in Figure 2(c1), Wu et al. combined hydrothermally synthesized rGO/Fe_3_O_4_ 3D porous networks with silicone rubber for dynamic smoke systems [44]. As shown in Figure 2(c2, c3), the material achieves infrared heat attenuation in the range of 26–43 °C (1–3 mg/cm^3^) and a peak microwave absorption of −31.3 dB at 15.3 GHz (2.0 mm thickness).

These studies systematically demonstrate core technological pathways involving multidimensional architectures (3D networks/aerogels/films), multi-mechanism coupling (dielectric–magnetic loss/thermal buffering/electromagnetic interference), and multifunctional integration (broadband absorption/infrared stealth/environmental resilience), providing theoretical foundations and material solutions for multispectral-compatible stealth and complex operational adaptability in military equipment.

### 3.2. LIG-Based Broadband Absorbing Material

**Figure 3:**
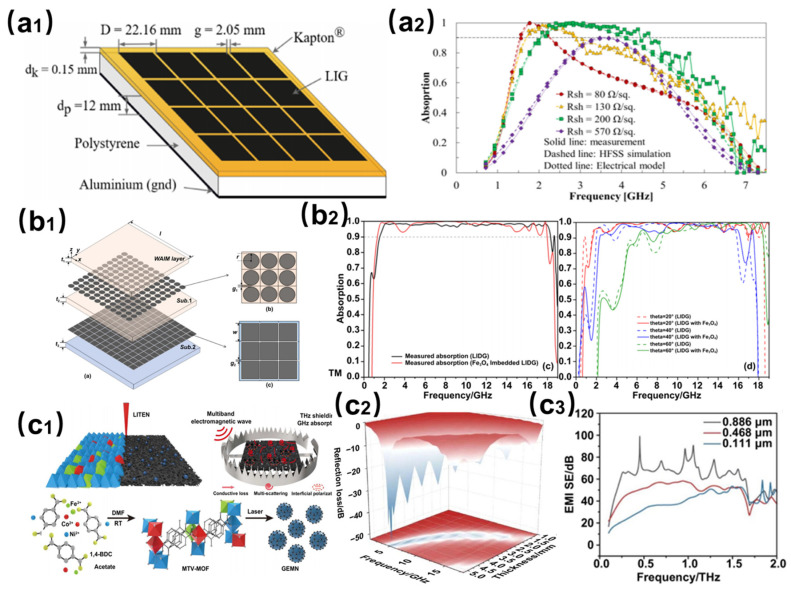
(**a1**) Laser processing method and design parameters of LIG-based single-layer frequency selective surface (FSS). (**a2**) Characterization of electromagnetic interference (EMI) shielding performance of FSS [45]. (**b1**) Laser-induced strategy for the synthesis of N/S co-doped graphene metamaterials with embedded magnetic nanoparticles. (**b2**) Electromagnetic absorption properties of metamaterials [46]. (**c1**) Laser additive manufacturing of graphene encapsulated magnetic nano-alloy heterostructures. (**c2**) Analysis of electromagnetic absorption properties of graphene encapsulated magnetic nano-alloy heterostructures. (**c3**) Analysis of electromagnetic shielding properties of graphene-encapsulated magnetic nanoalloy heterostructures [47].

Recent advancements in broadband microwave absorption materials highlight the pivotal role of laser-induced graphene (LIG) in achieving broadband performance through tailored structural designs and material doping strategies. As shown in Figure 3(a1), Houeix et al. pioneered a single-layer frequency-selective surface (FSS) based on LIG, fabricated via a one-step laser photothermal process on polyimide substrates [45]. As shown in Figure 3(a2), this approach synthesizes periodic resistive patterns directly on the substrate, enabling ultrathin (12 mm, 0.068λ_max_) and highly efficient low-frequency absorption (>90% within 1.69–2.91 GHz). The flexibility of structural design allows frequency response tuning by adjusting material properties, offering lightweight solutions for radar stealth and compact electronic devices. As shown in Figure 3(b1), to address the demand for low-frequency broadband absorption, Huang et al. developed a nitrogen/sulfur co-doped graphene metamaterial embedded with magnetic nanoparticles using laser direct writing [46]. As shown in Figure 3(b2), by precisely controlling sheet resistance uniformity (deviation <6%), the material achieved passive high absorption (>97%) across an ultra-wide bandwidth (1.56–18.3 GHz) under incident angles of 0–40°, demonstrating potential for aviation equipment and 5G base station electromagnetic interference (EMI) suppression.

For multiband compatibility in 6G communications, as shown in Figure 3(c1), Zhang et al. proposed a breakthrough strategy—ultrafast laser-induced thermochemical transformation and nanoalloy encapsulation (LITEN)—to fabricate graphene-encapsulated magnetic nanoalloys (GEMNs) [47]. As shown in Figure 3(c2,c3), this material integrates low-frequency absorption (reflection loss of −50.6 dB at 4.98 GHz in the C-band) and terahertz (THz) shielding (average effectiveness of 55.47 dB over 0.1–2 THz), providing a novel pathway for 6G signal management and THz radar stealth. These technologies collectively highlight the advantages of laser-based approaches in material customization through material removal, additive manufacturing, modification, and induced synthesis: LIG-FSS achieves selective low-frequency absorption with ultrathin monolayers; magnetic-doped metamaterials expand bandwidth and angular adaptability through resistance uniformity control; and GEMNs bridge GHz-THz functionalities via nanoalloy encapsulation.

**Figure 4:**
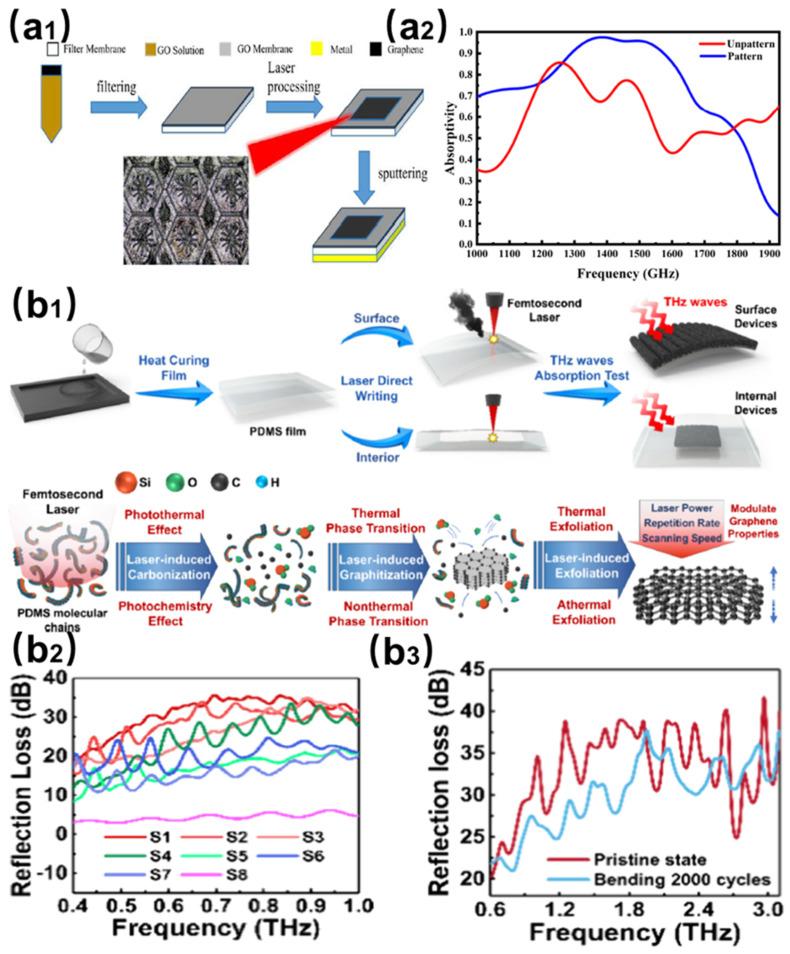
(**a1**) Preparation strategy of femtosecond laser-reduced graphene oxide (rGO) metamaterials. (**a2**) Terahertz wave absorption properties of rGO metamaterials [48]. (**b1**) Processing routes for femtosecond laser-induced PDMS substrate graphene (surface and internal). (**b2**) Terahertz absorption properties of femtosecond laser-induced PDMS substrate graphene. (**b3**) Comparison of terahertz absorption properties before and after bending 2000 times [49].

As shown in Figure 4(a1), Wu et al. constructed a three-layer metamaterial structure consisting of graphene oxide/dielectric spacer/gold substrate by reducing graphene oxide using a femtosecond laser (1030 nm, 55 mW, 1 MHz, 10 mm/s) [48]. Figure 4(a2) shows that the design achieves more than 90% broadband absorption in the 1.29–1.58 terahertz range, with 98.5% peak absorption at 1.44 THz. CST simulations elucidate the basic electromagnetic resonance coupled absorption mechanism. As shown in Figure 4(b1), You et al. pioneered a breakthrough strategy for the synthesis of 3D porous graphene by femtosecond laser. The microstructure of the material was synergistically optimized by precisely controlling the repetition frequency of the laser beam and, hence, the thermal and non-thermal interactions with the PDMS film [49]. As shown in Figure 4(b2,b3), the resulting flexible tunable terahertz absorbers demonstrated broadband absorption performance (80–99%) across 0.4–3.1 THz, with maximum reflection loss reaching 41.6 dB. Furthermore, the integrated encapsulation process significantly streamlined device fabrication. In contrast to conventional fabrication processes for electromagnetic absorbing materials (e.g., solid-state reactions, sol–gel methods, and chemical vapor deposition), these advancements collectively demonstrate the technological merits of laser microprocessing in developing next-generation absorbers for terahertz and microwave regimes, showcasing promising potential in stealth technologies and intelligent electronic systems.

**Figure 5:**
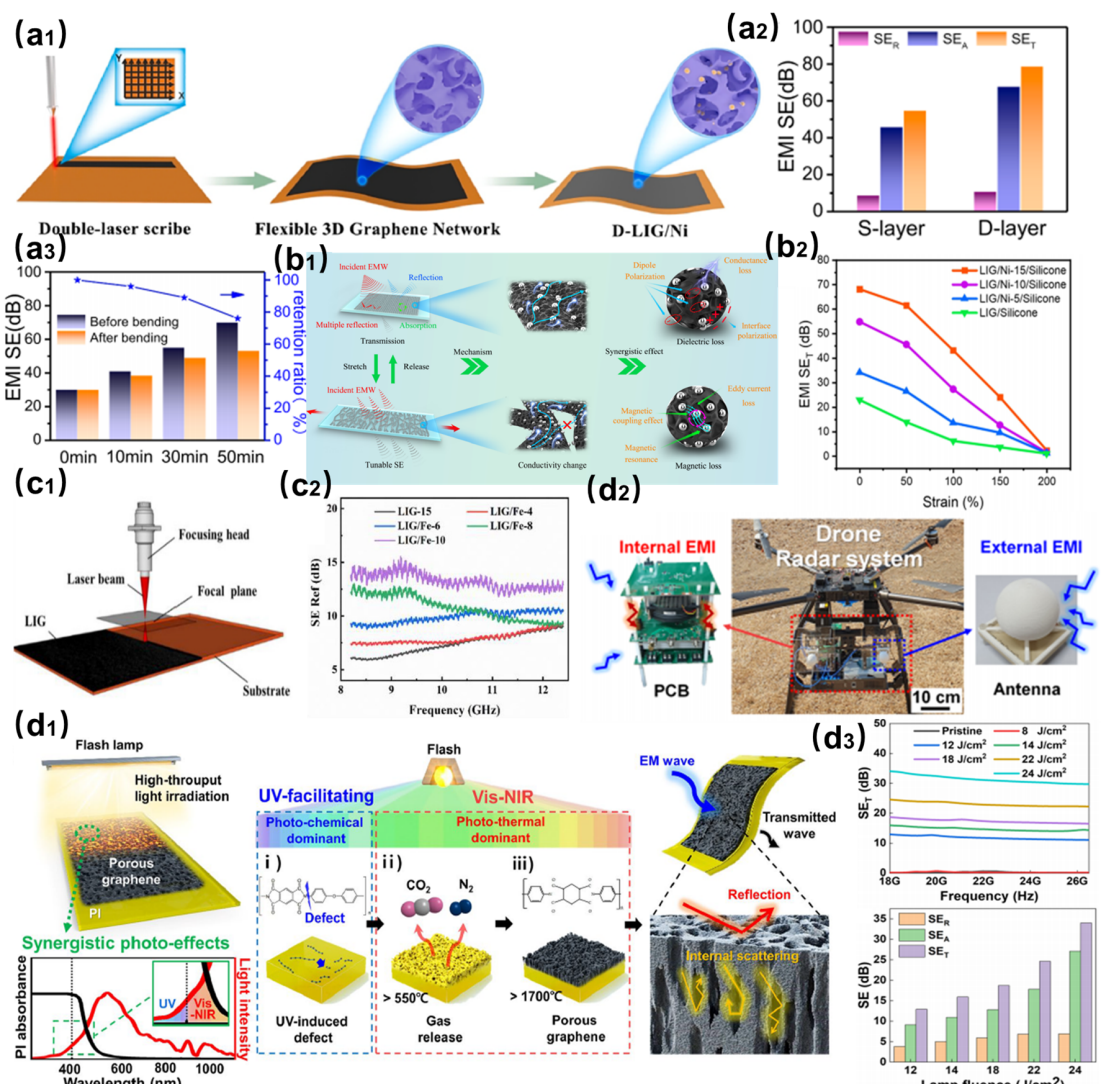
(**a1**) Preparation flow of LIG doped with magnetic nickel nanoparticles. (**a2**) Electromagnetic interference (EMI) shielding properties of LIG doped with magnetic nickel nanoparticles. (**a3**) Comparison of electromagnetic shielding properties before and after bending [36]. (**b1**) Preparation process of LIG-doped nickel-doped stretchable EMI shielding material using silica gel as the carrier. (**b2**) Electromagnetic shielding properties of LIG-doped nickel materials [50]. (**c1**) Preparation process of LIG-doped Fe_3_O_4_ nanoparticles. (**c2**) Electromagnetic shielding properties of LIG-doped Fe_3_O_4_ nanoparticles [51]. (**d1**) Preparation of flash-induced graphene (FPG) by pulsed photon irradiation. (**d2**) FPG for electromagnetic shielding of small Unmanned Aerial Vehicles. (**d3**) Electromagnetic shielding performance of FPG [52].

As shown in Figure 5(a1), Yin et al. constructed a polyimide-based flexible composite film by integrating three-dimensional porous laser-induced graphene (LIG) with magnetic nickel nanoparticles [36]. As shown in Figure 5(a2), the synergistic effects of dielectric loss from the LIG framework, multiple internal reflections, and magnetic loss from Ni NPs enabled the composite to achieve a shielding effectiveness (SE) of 55 dB and an absorption ratio of 82% in the X-band (8.2–12.4 GHz). Figure 5(a3) shows that this film displays excellent flexibility, retaining 89% SE after 500 bending cycles, making it a reliable solution for curved electronic devices. As shown in Figure 5(b1), Ge et al. developed a highly stretchable conductive framework by embedding Ni-doped LIG (LIG/Ni) into silicone [50]. As shown in Figure 5(b2), by optimizing LIG scanning trajectories and Ni NPs deposition parameters, ordered conductive pathways were engineered. Under 200% strain, the conductivity decreased to 1.07 S/cm (SE reduced to 2.33 dB), while strain release restored conductivity to 63.6 S/m (SE: 68.12 dB), achieving a wide SE tunability range (ΔSE > 65 dB) and cyclic stability. This innovation provides a novel strategy for dynamic electromagnetic protection in stretchable electronics.

To address the inherent limitations of LIG’s conductivity and substrate dependency, as shown in Figure 5(c1), Yu et al. pioneered a defocused laser process using polybenzoxazine precursors to fabricate highly conductive LIG [51]. As shown in Figure 5(c2), the resulting LIG exhibited an SE of 24.8 dB in the X-band at 68 μm thickness. The further incorporation of Fe_3_O_4_ nanoparticles enhanced the SE to 32.7 dB at 53 μm thickness. A rapid quenching–peeling (RQP) strategy was developed to detach LIG films from polymer substrates, yielding freestanding LIG membranes with superior lightweight performance (absolute SE surpassing most carbon-based materials) and adaptability for applications such as Joule heating devices. As shown in Figure 5(d1), Lee et al. synthesized porous flash-induced graphene (FPG) with hollow pillar structures via pulsed flash irradiation [52]. As shown in Figure 5(d2,d3), the FPG exhibited ultralow density (0.0354 g/cm^3^) and exceptional absolute SE (1.12 × 10^5^ dB·cm^2^/g). Practical validation in drone radar systems and wearable devices confirmed its efficacy in reducing specific absorption rate (SAR), establishing a new paradigm for EMI shielding in mobile scenarios.

### 3.3. LIG-Based Metasurface and Smart Cells

**Figure 6:**
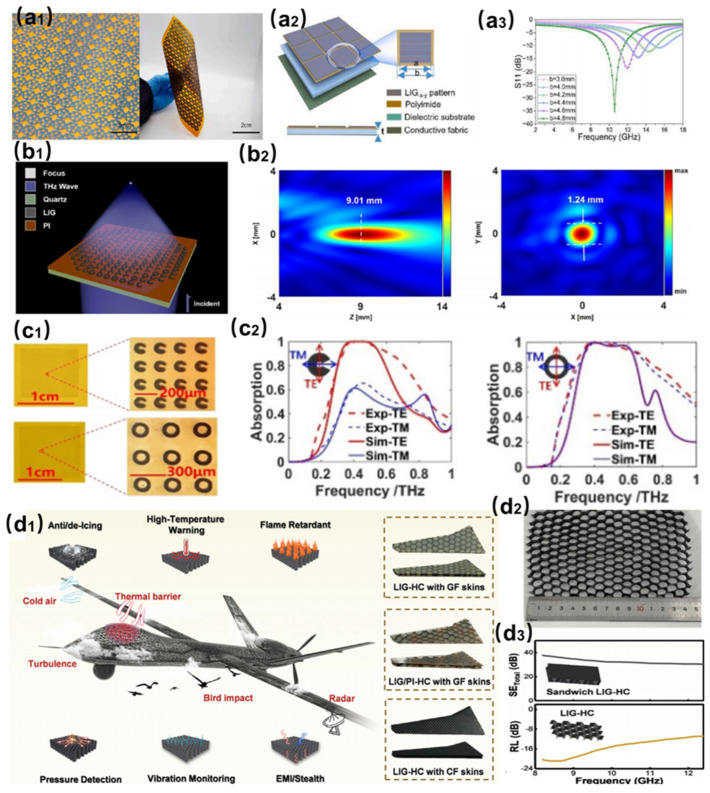
(**a1**) Fabrication process of LIG metasurfaces. (**a2**) Structural composition of LIG metasurfaces. (**a3**) Electromagnetic modulation properties of LIG metasurfaces [53]. (**b1**) Design and fabrication of LIG-based terahertz optical metasurfaces. (**b2**) Performance of LIG-based terahertz optical metasurfaces [54]. (**c1**) Fabrication process of LIG-based metasurface absorbers. (**c2**) Performance of LIG-based metasurface absorbers [55]. (**d1**) Fabrication and multifunctional applications of LIG intelligent honeycomb structures. (**d2**) Physical diagram of LIG intelligent honeycomb structures. (**d3**) Electromagnetic absorption and shielding properties of LIG intelligent honeycomb structures [56].

In recent years, laser-induced graphene (LIG)-based metasurfaces and intelligent honeycomb structures have demonstrated remarkable potential in microwave absorption, terahertz (THz) modulation, and multifunctional smart architectures, emerging as cutting-edge materials to address electromagnetic pollution, THz technology bottlenecks, and complex engineering demands. In the field of microwave metasurfaces, as shown in Figure 6(a1), Chen et al. optimized LIG metasurface structures by tuning laser parameters (power density, engraving rate) to develop flexible metamaterial absorbers (MAs) [53]. Figure 6(a2) shows the structural components of the absorber. As shown in Figure 6(a3), these absorbers achieved a minimum reflection loss (RL) of −39.7 dB and an effective absorption bandwidth (EAB) of 5.5 GHz at a thickness of only 1.1 mm, with a maximum EAB of 12.1 GHz (RL −18.9 dB). Multidirectional radar cross-section (RCS) simulations further validated their application potential in satellite electromagnetic protection. Such designs overcome the limitations of traditional absorbers in achieving high performance at ultrathin thicknesses by balancing impedance matching and electromagnetic loss mechanisms. In THz metasurface modulation, LIG has been exploited for low-cost, rapid fabrication of THz devices due to its high conductivity. As shown in Figure 6(b1), Wang et al. fabricated a 15 mm × 15 mm LIG-based THz metasurface with 30 μm resolution in 34 s using a nanosecond laser and telecentric scanning system [54]. The results in Figure 6(b2) show a resolution of 30 μm and the optical parameters satisfy the terahertz wave modulation requirements. As shown in Figure 6(c1), Dong et al. proposed a rapid, printable, and cost-effective metasurface absorber based on LIG technology [55]. As shown in Figure 6(c2), both metasurfaces achieved maximum absorption rates of 99.3% and 99.9% at resonant frequencies under incident angles of ±55°, with a single device (1 × 1 cm) fabricated in merely 11 s, offering efficient solutions for THz communication and modulation.

Furthermore, LIG exhibits unique advantages in intelligent structural design. As shown in Figure 6(d1), Gao et al. constructed smart LIG-based honeycombs (LIG-HCs) by the layer-by-layer stacking of LIG and adhesives, enabling tunable unit sizes, shapes, and patterned graphene distributions [56]. Figure 6(d2) shows the physical LIG-HCs, which are lightweight, low-cost, and easy to fabricate. As shown in Figure 6(d3), the systematic optimization of anisotropic mechanical, piezoresistive, and electromagnetic properties (e.g., shielding efficiency > 30 dB) was achieved through process–performance correlation studies. A representative application, the LIG-HC aircraft wing model, integrates multifunctional capabilities such as anti-/de-icing, high-temperature warning, flame retardancy, pressure/vibration monitoring, and electromagnetic stealth, highlighting its potential for integrated aerospace applications.

## 4. Challenges and Outlook

We systematically elucidated the multidimensional influence mechanisms of electrical conductivity, magnetic permeability, defect-state density, and micro–macrostructural characteristics on the electromagnetic shielding performance of LIG-based composites. As shown in Table 1, LIG-based composites offer significant advantages in terms of electrical conductivity, magnetism, structural flexibility, and absorption efficiency over conventional electromagnetic shielding systems. Extensive studies have demonstrated that the cross-scale synergistic design of electrical/magnetic properties is pivotal to overcoming current bottlenecks in electromagnetic shielding performance. By introducing conductive reinforcements (e.g., metallic nanoparticles, MXenes) and magnetic components (e.g., transition metal oxides) while preserving the inherent three-dimensional conductive network of LIG, a magneto-dielectric dual-loss synergistic effect can be constructed. This “conductive skeleton and magnetic node” structural design effectively enhances absorption peak intensity and broadens absorption bandwidth through the coordinated action of interfacial polarization effects and eddy current loss.

Despite the notable advantages of laser-induced graphene (LIG) technology in broadband electromagnetic management (microwave to terahertz regimes) through cross-scale microstructure control and macro-scale gradient/multilayer designs, its practical implementation faces multidimensional challenges:

(1) Technical Bottlenecks: Balancing LIG’s intrinsic flexibility/ultrathin characteristics with interfacial compatibility in composite systems while suppressing process fluctuations and interfacial defects during scalable manufacturing.

(2) Performance Limitations: Insufficient dynamic tuning range and environmental adaptability (extreme temperature/humidity, mechanical stress).

(3) Industrialization Barriers: Laser rapid in situ induction process reduces costs but necessitates resolving stability–cost trade-offs in multi-material integration (magnetic nanoparticles, phase-change materials).

Future advancements require synergistic strategies:

(1) Multiphysics-Coupled Design: Precise laser parameter modulation to tailor crystallographic defects and magnetic/dielectric filler distribution, enhancing polarization loss synergies. Hybridizing FSS with metamaterial architectures for full-spectrum coverage and wide-angle impedance matching.

(2) Intelligent Responsive Systems: Integrating thermally/electrically responsive materials (e.g., topological insulators) with AI-driven in situ monitoring to construct adaptive shielding systems for dynamic frequency-band optimization (5G/6G compatibility) and environmental robustness.

(3) Eco-Manufacturing Paradigms: Developing low-temperature laser processing/self-assembly hybrid techniques to reduce energy consumption and toxic solvent dependence while enhancing production rates and consistency.

This roadmap will accelerate the transition of LIG-based materials from laboratory prototypes to aerospace stealth coatings and wearable electronic armor, ultimately achieving next-generation electromagnetic shielding systems with adaptivity, full-spectrum coverage, and environmental resilience.

## Figures and Tables

**Table 1:** Comparison of LIG-based composites with conventional electromagnetic shielding systems.

Characteristics	LIG-Based Composites	Conventional Electromagnetic Shielding Systems (e.g., Metal, Ferrite, etc.)	Summary of LIG Advantages
Conductivity	High conductivity (10^3^∼10^4^ S/m), porous structure to enhance the conductive network [57].	Metals (e.g., copper, aluminum) have high electrical conductivity (10^7^ S/m), but are easily oxidized and heavy [58,59].	Lightweight, corrosion-resistant, optimized conductive network through porous structure for high-frequency applications.
Magnetism	Controllable magnetism can be achieved by compounding magnetic materials (e.g., Fe_3_O_4_, nickel particles) without significant weight gain [36,60].	Dependent on ferrite or metal magnetic materials, high magnetic properties but high density, complex processing [61,62].	Magnetically adjustable for flexible/multifunctional integration scenarios.
Structural flexibility	Highly flexible; can be made into films, fabrics, or 3D structures; adapts to curved surfaces and dynamic deformations [63,64].	Metals or rigid composites (e.g., aluminum foil, ferrite sheets) are difficult to bend and require complex processing to achieve a specific shape [65,66].	In situ patterning of high-precision complex structures and support for customized designs.
Electromagnetic absorption/shielding efficiency	Porous structure enhances multiple reflections of electromagnetic waves and dielectric loss, absorption dominated shielding (SE > 30 dB, absorption > 90%) [25,36].	High shielding efficiency, but reflection shielding accounted for a high proportion; easy to cause secondary electromagnetic pollution [67,68,69].	More environmentally friendly absorption mechanism, reduced reflection pollution, excellent high-frequency absorption performance (e.g., 5G, terahertz band).
Comprehensive performance	Lightweight, high conductive/dielectric loss, customizable multifunctional integration (e.g., thermal conductivity, sensing) [56,70].	Single-function, high-density materials limit their use in aerospace and other applications [71].	Breaking through the limitations of traditional materials to meet the needs of modern electronic devices for lightweight, flexibility, and efficient absorption.

## Data Availability

No new data were created or analyzed in this study.

## Abbreviations

The following abbreviations are used in this manuscript:

LIG	laser-induced graphene
EMI	electromagnetic interference
SE_R_	reflection loss
SE_A_	absorption loss
SE_T_	total shielding effectiveness
SE_M_	multiple reflection loss
FSS	frequency-selective surfaces
EAB	effective absorption bandwidth
LIG-HC	LIG-based honeycombs
MAs	metamaterial absorbers
NPs	nanoparticles
PDMS	polydimethylsiloxane
CST	cell signaling technology
GEMN	graphene-encapsulated magnetic nanoalloy
LITEN	laser-induced thermochemical transformation and nanoalloy encapsulation
